# Female Genital Mutilation: Health Consequences and Complications—A Short Literature Review

**DOI:** 10.1155/2018/7365715

**Published:** 2018-07-10

**Authors:** Elliot Klein, Elizabeth Helzner, Michelle Shayowitz, Stephan Kohlhoff, Tamar A. Smith-Norowitz

**Affiliations:** ^1^Department of Pediatrics, Division of Infectious Diseases, State University of New York Downstate Medical Center, Brooklyn, NY 11203, USA; ^2^Department of Epidemiology and Biostatistics, School of Public Health, State University of New York Downstate Medical Center, Brooklyn, NY 11203, USA

## Abstract

Female genital mutilation (FGM) is a procedure performed on women in developing countries and is underreported; it involves cutting or altering the female genitalia. The health consequences of FGM include bacterial and viral infections, obstetrical complications, and psychological problems. In this study, we report FGM societal importance, ramifications, classifications, cultural significance, prevalence, complications, implications, and treatment. Although efforts have been made to eradicate FGM, the dynamics that perpetuate the practice have societal roots. Intervention methods to promote change from within the community are necessary for successful eradication of the practice. For prevention, further studies are needed to develop programs that raise awareness.

## 1. Introduction

Female circumcision (FC) or female genital mutilation (FGM) describes practices that manipulate, alter, or remove the external genital organs in young girls and women [1]. The procedure is performed using a blade or shard of glass by a religious leader, town elder, or a medical professional with limited training. In about 15% of cases, infibulation, the most severe form of FGM, involves the removal of the labia and the suturing together of the vulva; this practice may place the victim's life at risk [1]. In contrast to male circumcision, the procedure produces no known health benefits and is not performed for medical reasons [2]. FGM is widely recognized as a procedure that violates a person's human rights, as well as increasing their risk for health complications [2]. The aim of the present study was to compare literature sources regarding the practice and negative outcomes of FGM as well as explore the phenomena perpetuating the custom.

## 2. Methods

PRISMA guidelines were followed according to the studies of Moher et al. [3] and Shamseer et al. [4]. Our review question sought to investigate the consequences of FGM, its cultural and social dynamics, and possible approaches and obstructions to eradication. 42 articles published between the years 1994 and 2017 were reviewed. Database searches included PubMed, MEDLINE, Google Scholar, Web of Science, and EBSCOhost. In addition, the data from four international organizations that address FGM were reviewed for relevant information: specifically, the Population Reference Bureau (PRB), the United Nations Children's Fund, the United Nations Population Fund, and the World Health Organization (WHO).

No date restrictions were imposed, and all study types were explored including systematic reviews, cohort studies, case-control studies, case series, cross-sectional studies, case reports, and randomized controlled studies to encompass qualitative research and the qualitative element of diversified methods. As FGM is not an overtly researched field, especially within the United States, it was important to explore all sources of information in order to generate the best understanding of FGM procedures. Searches were refined to articles written in English and published in a peer-reviewed or organizational journal in order to retain the integrity of the informational sources. The search strategy incorporated subject headings and text words relating to FGM. We included the four alternative classifications for the procedure, including mutilation, circumcision, excision, and cutting. We did not apply any methodological search filters in order to maximize sensitivity. This strategy was applied to all databases reported above.

Results from database searches underwent titles and abstracts screening by two separate reviewers for relevance and inclusion. Any discrepancies were submitted and resolved by a third reviewer who examined the pertinence and concerns of the two initial reviewers. Studies considered for inclusion were then retrieved as full-text articles. Final decisions about inclusion were made by the authors, and reasons for exclusion were documented. Using a specifically developed piloted data extraction form, information from all reports was recorded (e.g., details of the phenomenon of interest, population, context, study methodology, methods, and outcomes of significance). For each outcome, we aimed to extract the relevant statistical results, which included prevalence incidence rates, relative risk, odds ratio, and chi-squared goodness-of-fit tests. No assumptions or simplifications were made other than those listed by the authors in their original studies.

The risk of bias was assessed in all studies by three independent review authors. Each reviewer then recorded his or her findings on a separate “Bias Assessment Form.” Overlapping concerns of bias were then selected for final review and incorporated into the literature review. The concerns wholly addressed bias at the study level. For any conflicting information found within sources, data from organizations conducting research “on the ground” (i.e., UNICEF, WHO, and PBR) took precedence as their data were assumed to be more recent and accurate. No combining of results was performed, and all data were portrayed independently.

## 3. Results: Study Selection and Characteristics

### 3.1. Societal Importance

FGM is performed in developing countries with the most occurrences reported in sub-Saharan Africa, the Middle East, and Asia. These countries have many FGM victims, as the procedure produces a three-pronged platform that makes eradication difficult. FGM has deep sociological roots that create societal norms in order for families to be accepted by the communities. The social conventions place pressure on parents to perform FGM on their daughters in order to prepare them for marriage and adulthood. Its cultural significance leads to the notion that it maintains girls' chastity, preserves fertility, improves hygiene, and enhances sexual pleasure for men. FGM is utilized as an initiation rite of passage to womanhood and aims to ensure premarital virginity and marital fidelity by reducing her desire for extramarital sexual acts. When the vaginal opening is altered to create a smaller orifice, the fear of opening it further discourages extramarital sexual intercourse [5]. Parents and religious leaders enforce circumcision throughout their communities in order to ensure the next generations of children maintain tradition. The combination of these aforementioned factors creates a dynamic that renders FGM a public health concern that requires cultural competence to address [6].

### 3.2. Cultural Significance

The cultural and traditional components of FGM vary between ethnic enclaves [7]. The procedure is routinely carried out between the ages of six and eight with a few cultures preferring to cut at birth, menarche, or before marriage [8]. Mutilation is more often undergone alone, but can occur in groups, using same instruments on more than 40 women [9]. The procedure is almost always performed in a ceremonial manner accompanied by music, food, and gifts. The operators can range from “circumcisers” (religious leaders) with no medical training to midwives and birth attendants. The tools used include knives, clippers, scissors, or hot objects [10]. A sterile environment is not feasible to attain in the cast majority of cases, and no medical anesthetics are available; the wound is sewed with crude instruments such as thorns. When infibulation takes place, thorns or stitches may be used to hold the two sides of the labia majora together and the legs may be bound together for up to forty days [11, 12]. The healing process is aided by ointments and compounds made of herbs, milk, eggs, ashes, sugar, or animal excrement, which is thought to facilitate healing.

Girls undergoing the procedure have varying degrees of knowledge about what will happen to them. Girls are encouraged to be brave and not to cry during the procedure lest it will bring shame onto their family [13]. Only women are allowed to be present at the ceremony. In some cultures, girls will be told to sit beforehand in cold water to numb the area and reduce the likelihood of severe bleeding [13]. However, no steps are taken to reduce the pain [13].

### 3.3. Ramifications of FGM

Currently, female circumcision is practiced in 30 countries throughout Africa and the Middle East with an estimated 200 million women worldwide currently infibulated [5]. Despite global and regional attempts at ending genital mutilation by law and intervention methods, the custom has persisted through the ages. Although it is recognized internationally as an infringement on human rights, the multifaceted dynamic makes it difficult to eradicate [14]. The ramifications of FGM affect the girl for the rest of her life and result in many health problems (i.e., extended bleeding, problems with urination, cysts, infections, and complications during childbirth). Aside from health-related, ethical, and moral consequences of FGM, it has been estimated by the World Health Organization (WHO) that the annual cost of obstetric complications is more than $3.7 million [15]. However, rationalization of genitalia mutilation persists; the people conducting the procedure do not believe they are doing harm. The eradication of FGM as a public health initiative is imperative to ensuring that newborn females and youth do not undergo this traumatic ordeal. Moreover, immigrant populations arriving in developed countries, particularly the United States (U.S.), present a particular obstacle in the full-global abolition of female genital mutation as many seek to continue their cultural traditions [16, 17].

Since 1997, conducting or practicing female circumcision on a minor in the U.S. is considered a Class E felony [17]. Despite the obvious efforts to prevent FGM from occurring in the U.S., it remains a significant injurious tradition that may be carried out by family members in obscure locations. In some instances, parents fly their daughters back to their homeland to have the procedure before returning to seek better medical care [11]. Most American girls and women at risk of having a FGM procedure live in cities or suburbs of large metropolitan areas [11]. In particular, the tristate New York City area has an estimated 65,893 women who are at risk with more than 21,737 of them under the age of 18 [11]. Additionally, girls and women who have had the procedure prior to migrating may later present with varying degrees of complications. Many of these complications arise during pregnancy and childbirth. A third category of women seek out medical care in order to have the circumcision process reversed in a surgical procedure known as defibulation [18].

The complexity of FGM in its relation to urban and immigrant health is comprised of a combination of concerns that center on gender equality, religious freedom, cultural traditions, and societal norms [19]. Therefore, maintaining this tradition remains of utmost importance to many individuals whose region once practiced it. These issues form a dynamic that thrives within immigrant communities that make it increasingly difficult to eradicate the procedure [19].

### 3.4. FGM Classifications

The World Health Organization (WHO) classifies the mutilation of the female genital into four distinct categories [5]. Three of the four categories are further broken down into subcategories that classify the specific type of mutilation that was performed. Type I is known as clitorodectomy and includes any procedure that totally removes the clitoris and/or the prepuce [6]. Type Ia is the removal of the clitoris hood or prepuce only while Type Ib includes the removal of both the clitoris and the prepuce [6]. Type II, or excision, is the partial or total removal of the labia minora unrelated to any mutilation performed on the labia majora. Type IIa includes the removal of the labia minora only. Type IIb is the removal of the labia minora and the partial or total removal of the clitoris [6]. Type IIc involves the removal or the clitoris, labia minora, and labia majora. Infibulation, or Type III, is the third category of mutilation procedures defined as the narrowing of the vaginal orifice with the sealing of the perineum by cutting and repositioning the labia minora and labia majora with or without the excision of the clitoris. Type IIIa references specifically procedures done with the removal and apposition of the labia minora, while Type IIIb includes procedures done with only the labia majora [6]. Type IV is a broad category that includes all other harmful procedures done without medical purpose to the female genitals. This includes any cutting, herbal treatments, or burns that alter or harm the patient's body [20].

## 4. Main Finding

### 4.1. Prevalence and Trends

**Figure 1:**
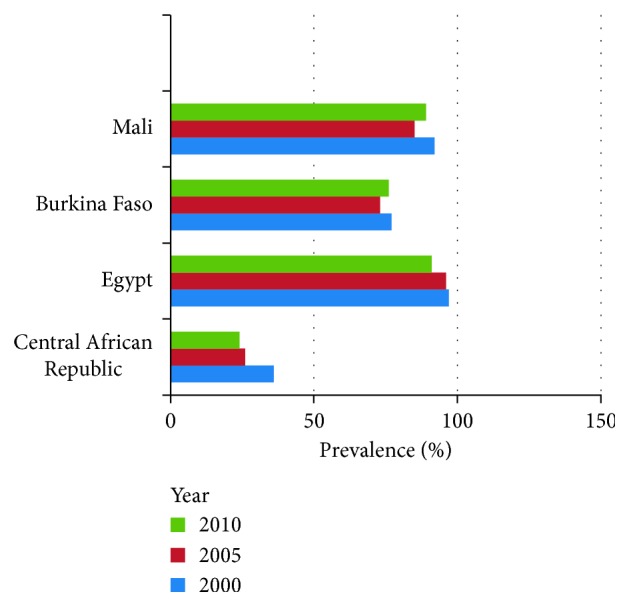
FGM 10-year prevalence trends. Data collected by the PRB showing prevalence rates over a 10-year period (2000–2010) in four countries (Mali, Burkina Faso, Egypt, and Central African Republic).

Female circumcision is practiced in many regions throughout Africa, Asia, and the Middle East. However, the highest prevalence rates are found within the Horn of Africa, the region containing the countries of Djibouti, Eritrea, Ethiopia, and Somalia [21]. Prevalence rates within this province are estimated to be as high as 99% [21]. Other areas throughout the African continent have varying prevalence rates ranging between 2% and 95% [21]. In the Middle East, Egypt contains the densest population of FGM victims with approximately 27.2 million women having undergone the procedure [22]. Within Asia, Malaysia, India, and Indonesia have prevalence rates that exceed 90% in some regions [23]. Worldwide, it is estimated that more than 200 million women have been cut and approximately 6,000 girls are circumcised every day [23]. More than 3 million girls are at risk for circumcision on the continent of Africa [21]. An estimated 3 million girls are subject to one of the four types of mutilations each year with more than 85% eventually having a medical complication, sometime in their life as a result [24]. The prevalence trends have shown mixed results over the past two decades with little, if any, decline [25].  Figure 1 displays data collected by the Population Reference Bureau (PBR) in which varied trends are seen for the years 2000, 2005, and 2010 [25]. In some instances, prevalence rates have dropped only to return to their previous levels.

**Figure 2:**
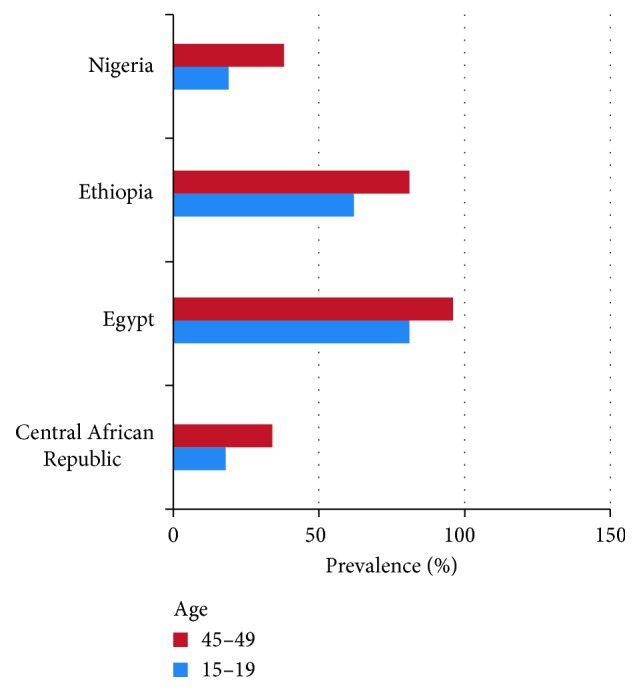
FGM prevalence among young women in 2014 exhibits decline. Data collected by the PRB in 2014 in younger women showing the prevalence of FGM among younger women (aged 15–19) in four countries (Mali, Burkina Faso, Egypt, and Central African Republic).

Although intervention programs have been introduced in Africa, Egypt, and much of Asia, the prevalence rates have not been significantly lowered [25]. Several countries, specifically the ones with more stabilized governments who have taken stances against mutilation, have begun to see a slight decline with the younger population indicating a possibility of eradication. As most girls undergo mutilation between the ages of 6 and 12, looking at the prevalence of girls currently aged between 15 and 19 and comparing them to those of women aged between 45 and 49 allow us to see a declining trend taking hold in some countries.  Figure 2 compares trends of both age groups with substantial differences [25]. This indicates a preliminary decline in the circumcision in younger women in these countries.

### 4.2. Consequences and Complications of FGM

The consequences of FGM have both physiological and psychological complications [26], including short- and long-term complications [26]. The method in which the procedure is performed may determine the extent of the short-term complications [13]. If the process was completed using unsterile equipment, no antiseptics, and no antibiotics, the victim may have increased risk of complications. Primary infections include staphylococcus infections, urinary tract infections, excessive and uncontrollable pain, and hemorrhaging [27]. Infections such as human immunodeficiency virus (HIV), *Chlamydia trachomatis*, *Clostridium tetani*, herpes simplex virus (HSV) 2 are significantly more common among women who underwent Type 3 mutilation compared with other categories [27]. As the short-term complications manifest, mortality risk increases because of the limited health care available in low-income economies. While data on the mortality of girls who underwent FGM are unknown and hard to procure, it is estimated that 1 in every 500 circumcisions results in death [21]. The belief that the procedure produces protective factors against sexually transmitted infections (STIs), much like male circumcision, was disproved in a case-control study conducted in Sudan [27]. After the area heals, victims suffer the long-term consequences of the abuse through both physiological and psychological complications and substantial complications during childbirth [28, 29].

One of the most common long-term complications is the development of keloid scar tissue over the area that has been cut [30]. This disfiguring scar can be a source of anxiety and shame to the women who had FGM [30]. Neuromas may develop because of entrapped nerves within the scar leading to severe pain especially during intercourse [31]. First sexual intercourse can only take place after gradual and painful dilation of the opening left after mutilation. In a study carried out in Sudan, 15% of women interviewed reported that cutting was necessary before penetration could be achieved [31]. Other side complications include cysts, haematocolpos, dysuria and recurrent urinary infections, and possible infertility [31]. Childbirth for infibulated women presents the greatest challenge, as maternal mortality rates are significantly higher because of complications that arise during labor. During delivery, infibulated women (i.e., genitals have been closed tighly) are cut in the perineum area so that the baby can be delivered safely. [26].

Posttraumatic stress disorder (PTSD), anxiety, depression, neuroses, and psychoses are common delayed complications that are associated with FGM [32–34]. In developing countries, these conditions regularly go unrecognized and if left untreated, may lead to mental concerns later in life.

### 4.3. Implications and Limitations

The implications of FGM include both psychological and social factors. Prior literature reported the association between female circumcision and maternal morbidity and birth outcomes [26]. Studies have shown maternal prolonged maternal hospitalization, low birth weight, prolonged labor, obstructed labor, and increased frequency of cesarean sections as outcome variables in order to determine the consequences of FGM. In a prospective cohort study (4800 consecutive pregnant women in their first trimester) from Kuwait University Hospital, it was reported that the prevalence of FGM was 38% (1842), with significant results found between Extended Maternal Hospital Stay (OR = 1.5, CI 1.1–2.0), Prolonged Labor (OR = 3.4 CI 1.4–2.8), increased frequency of C-sections (OR = 1.7, CI 1.2–2.0), Hep C infections (OR = 1.6, CI 1.1–2.0), Obstructed Labor (OR = 2.3, CI 1.3–2.5), and Infant Resuscitation (OR = 2.2, CI 1.0–3.3) [26]. Additionally, positive associations between FGM and psychiatric sequelae that included flashbacks, psychiatric disorders, anxiety disorders, and PTSD (OR = 24.6, CI 1.9–22.2) were reported [26]. These outcomes coincide with the existing literature that depicts the relationship between FGM and a host of negative health effects including obstetrical, gynecological, and fetal sequelae [26].

Nevertheless, several limitations exist within the study may explain large confidence intervals and insignificant results in the outcomes analyzed. The inclusion criterion for FGM patients was very broad and included any marking or change that was performed on the female genital organs. This allowed confounding variables such as self-impairment that had no impact on the vaginal orifice (i.e., scratching and piercings) to be included and lower the internal validity. Moreover, patients with FGM were given preferential treatment (i.e., extended hospital stays and extra examination time) that may have led to biases in the study [26].

### 4.3. Psychological Long-Term Health Consequences

In addition to the obstetrical, gynecological, and neonatal impacts of FGM, other long-term health consequences include psychological and psychosexual disorders. A study conducted at the University of Lagos, Nigeria (*N*=350), analyzed the psychosocial aspects of FGM [35]. The questionnaire captured the sociodemographic aspects of respondents and included an array of questions regarding the practice, belief, and eradication of FGM [35]. 76% (266) of respondents were circumcised with clitoridectomy being the most prevalent mutilation type (83.1%). Additionally, 78.6% of women reported that a nurse or a midwife was the operator of their FGM procedure. Christianity (69.1%) appeared to be the most common religion practiced although the presence of other religions was amongst those circumcised as well [35]. The most common perception on the commonality and acceptance of circumcision was cultural significance (86.6%). Furthermore, respondents indicated that the reduction of female sexuality (61.4%) followed by tradition and customs (14.9%) were the reasons for practicing FGM [35]. Only 7.7% of participants agreed that the practice should be continued. Although 76% of the responders were circumcised, 91.7% reported that they do not plan to circumcise their own daughters. Sexual ramifications of FGM provided that 62% of circumcised women reported pain during intercourse when compared to 4% of those who did not (*χ*^2^ = 83.848, *p* < 0.01) [36]. In addition, 60.5% of circumcised women reported fear when their spouse called for sex compared to 2.4% of those who did not (*χ*^2^ = 86.742, *p* < 0.01). Thus, the psychosocial effects may impact the sexual experience of FGM victims and affect their personal relationships [35].

### 4.4. Treatment

Few treatment options exist for victims of FGM [36, 37]. Psychological and emotional support is available from therapist and support groups that specialize in PTSD [36, 37]. These support groups are often located in urban areas or near ethnic enclaves that have high risk of FGM. In addition, defibulation, a surgical process that attempts to reconstruct the labia by undoing the initial mutilation, is available at specialty hospitals throughout the world. However, many times the procedure has mediocre results and can result in additional complications. Additionally, the cost of the surgery is not always covered by insurance, thereby causing a financial deterrent [36, 37].

Foldès et al. conducted a study at St. Germain Poissy Hospital, France, from 1998 to 2009, assessing the immediate and long-term outcomes of reconstructive surgery [36]. Employing a prospective cohort study design, they followed 2938 women who had been operated on, from surgery to one-year follow-up. Prior to surgery, all patients filled out a questionnaire about tier demographic characteristics and preoperative pain at the mutilation site [36]. Subsequently, patients underwent surgery to restore both clitoral anatomy and function. In addition, for infibulated patients, defibulation preceded surgery in order to restore vaginal function. Patients were all discharged two days following surgery, returned for a two-week follow-up, and told to return in a year's time; a follow-up rate of 29% was achieved [36].

## 5. Discussion and Interpretation

The current review demonstrates that the practice of FGM remains prevalent in certain countries, even though there may exist laws against FGM. The elimination of FGM has made little progress over the past decade [25]. This may be due to the fact that developed countries have difficulties understanding the cultural and religious dynamics that communities and ethnicities practice FGM. Although activist movements are beginning to form throughout Africa, utilization of an intervention method that understands the diverse cultural dynamics can increase the results by introducing positive social changes. Engaging community and religious leaders through helping them understand the need for change is imperative in generating a transformation within the culture. Communities need to develop, strengthen, and support specific actions directed at ending FGM [9, 38].

## 6. Conclusion

FGM has been associated with medical, sociocultural, and economic consequences [9, 38]. Elimination of FGM is possible through directing resources in an efficient manner. Targeted interventions can include cultural and ethnical proponents. Thus, future research should explore the effects of intervention strategies to prevent FGM.

## Abbreviations

FGM: Female genital mutilation
FC: Female circumcision
PRB: Population Reference Bureau.
